# Supplementary material for: Macromolecular sheets direct the morphology and orientation of plate-like biogenic guanine crystals
Source: Nat Commun. 2023 Feb 3;14:589. doi: 10.1038/s41467-023-35894-6 (PMC9898273; doi:10.1038/s41467-023-35894-6)
Supplement: Supplementary file 1 — Supplementary Information [file 41467_2023_35894_MOESM1_ESM.pdf]

Supplementary Information

Macromolecular Sheets Direct the Morphology and Orientation of Plate-like Biogenic Guanine Crystals

Avital Wagner[1], Alexander Upcher[2], Raquel Maria[2], Thorolf Magnesen[3], Einat Zelinger[4], Graça Raposo[5,6], Benjamin A. Palmer[1]*

[1]Department of Chemistry, Ben-Gurion University of the Negev, Beer-Sheba 8410501, Israel.

[2]Ilse Katz Institute for Nanoscale Science & Technology, Ben-Gurion University of the Negev, Beer-Sheba 8410501, Israel.

[3]Department of Biological Sciences, University of Bergen, Postbox 7803, Bergen N-5020.

[4]The CSI Center for Scientific Imaging, The Robert H. Smith Faculty of Agriculture, Food and Environment, The Hebrew University of Jerusalem, POB 12, Rehovot 7610001, Israel.

[5]Institut Curie, PSL Research University, CNRS, UMR144, Structure and Membrane Compartments, 75005 Paris, France.

[6]Institut Curie, PSL Research University, CNRS, UMR144, Cell and Tissue Imaging Facility (PICT-IBiSA), 75005 Paris, France.

*Corresponding author: bpalmer@bgu.ac.il

Inventory of Supporting Information

Supplementary Figures 1-8

Supplementary Movies 1-2

Supplementary References 1-8

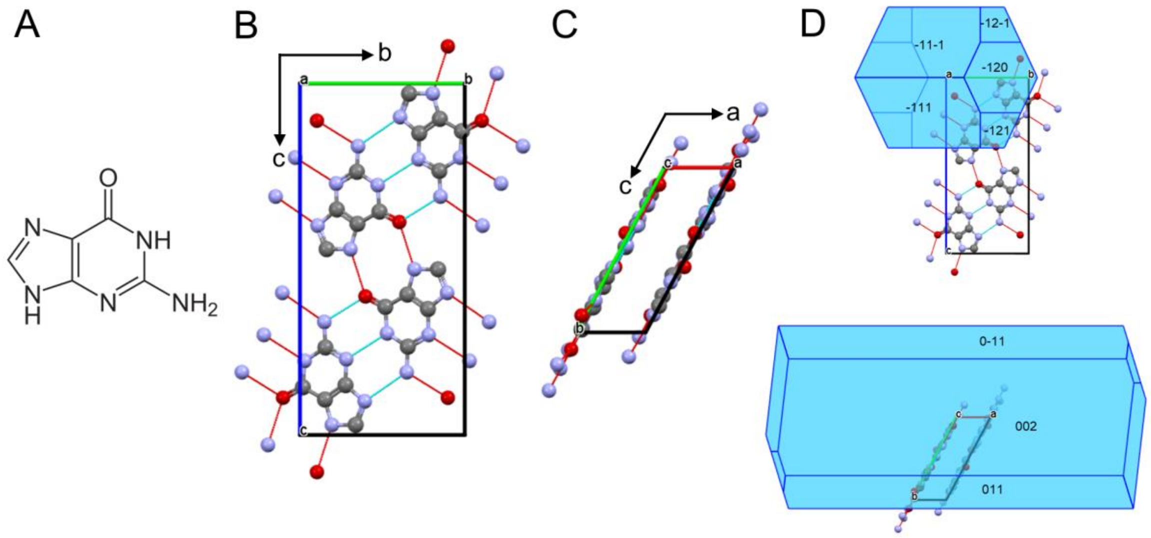

Supplementary Fig. 1. The *β*-guanine crystal structure. (A) Molecular structure of guanine. The crystal structure of *β*-guanine viewed perpendicular (B) and parallel (C) to the H-bonded layer[1]. (D) The corresponding thermodynamically stable BFDH morphology calculated by Mercury software.

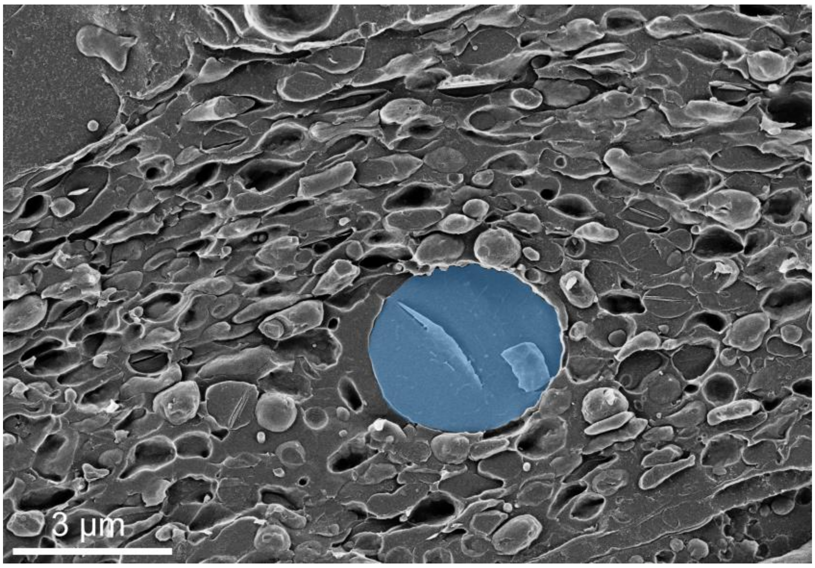

Supplementary Fig. 2. Cryo-SEM micrograph of the mirror region in a freeze fractured juvenile scallop eye. The nucleus (pseudo colored blue) of the iridophore is surrounded by ellipsoidal iridosomes of various stages, some containing partially formed guanine crystals.

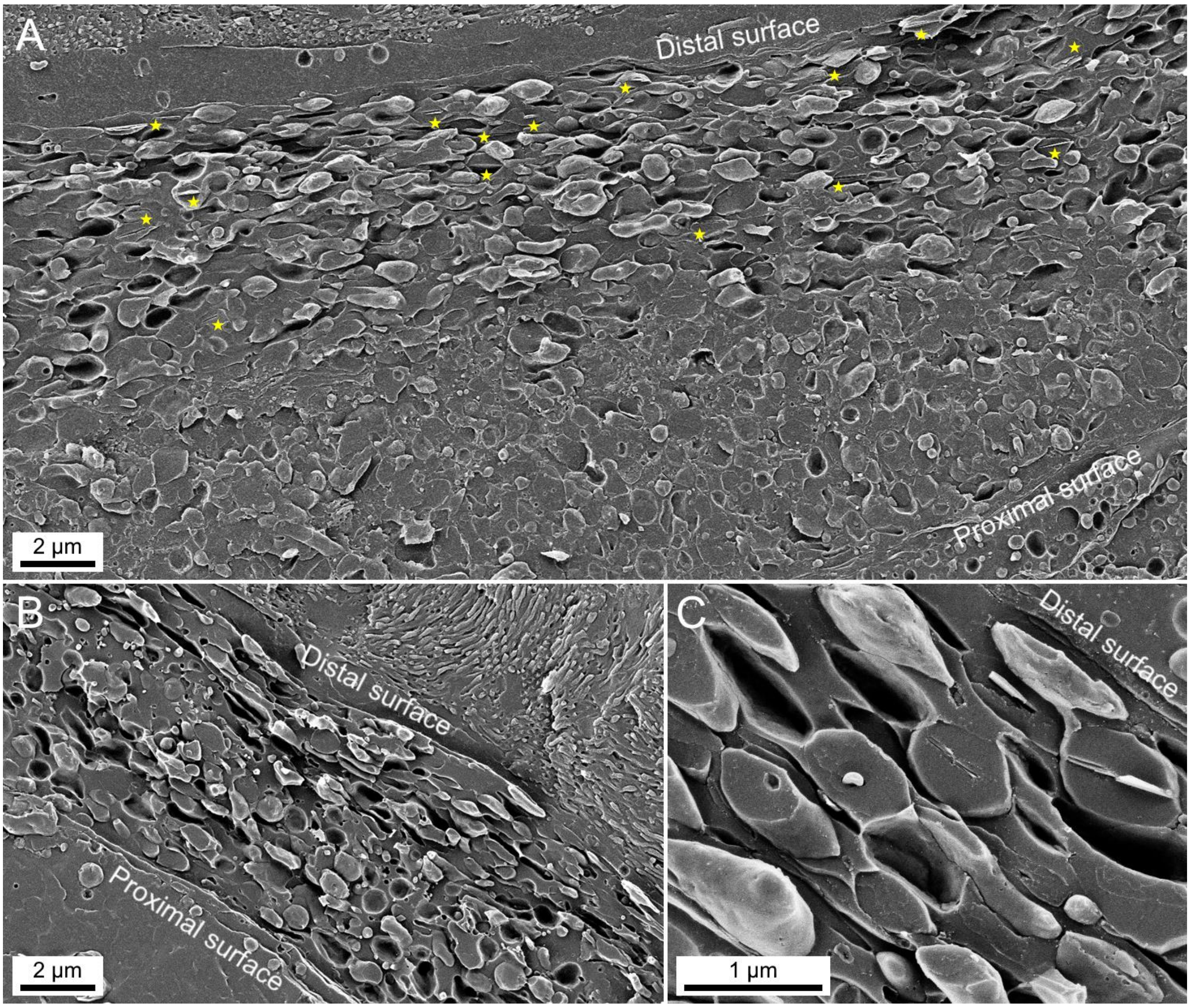

Supplementary Fig. 3. A few examples showing a special gradient of maturing iridosomes between the distal and proximal surfaces in the forming iridophore cell. (A) A central and (B) a side part of the mirror region showing both the distal and proximal surfaces of the region are seen. More mature elongated iridosomes are found near the distal surface, while less mature spherical iridosomes are found near the proximal surface. (C) A high magnification of Figure 1f in the manuscript showing a gradient of iridosome states. Yellow stars in (A) show location of immature guanine crystals.

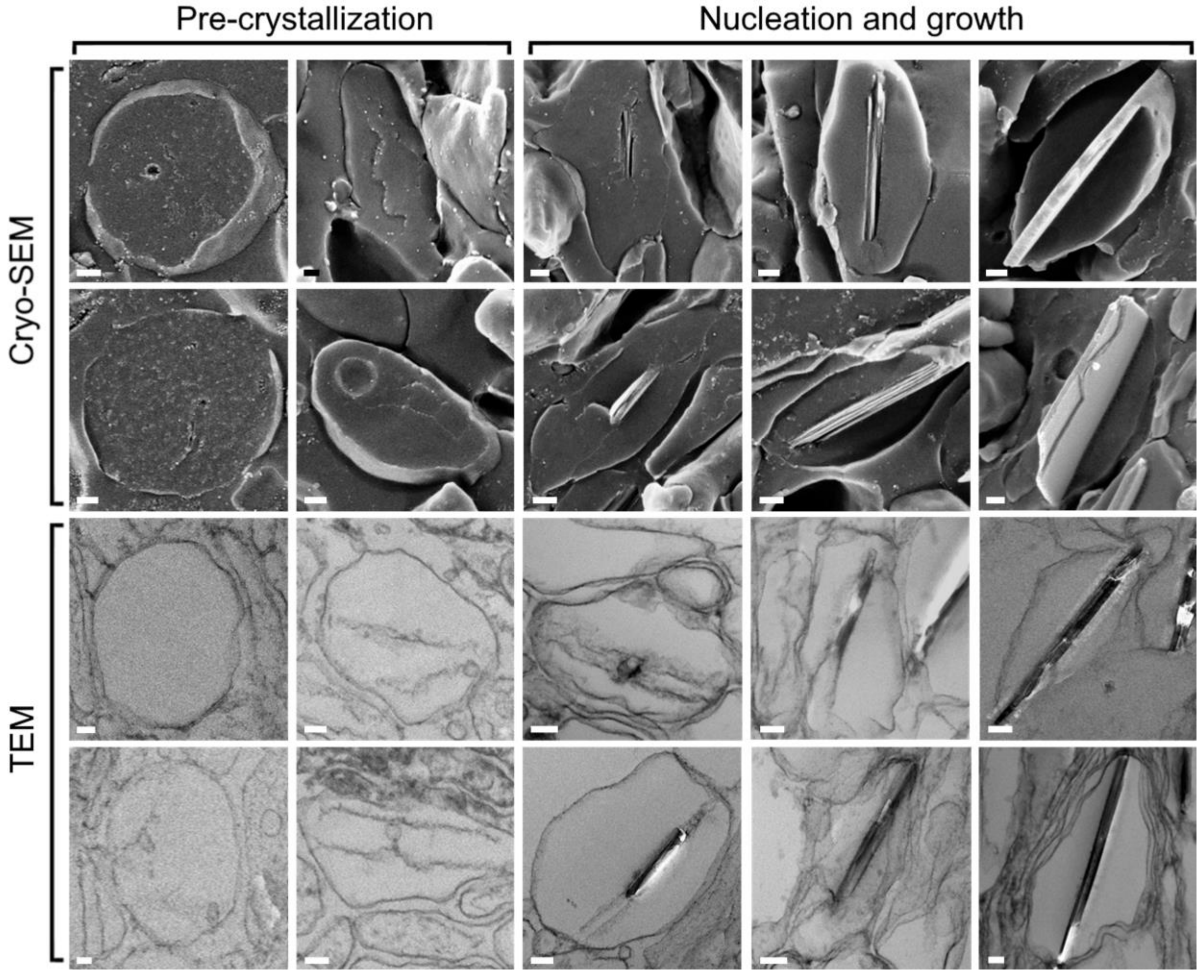

Supplementary Fig. 4. Additional examples of iridosomes at different stages of maturation (left to right) by cryo-SEM and TEM. Scale bars: 100 nm.

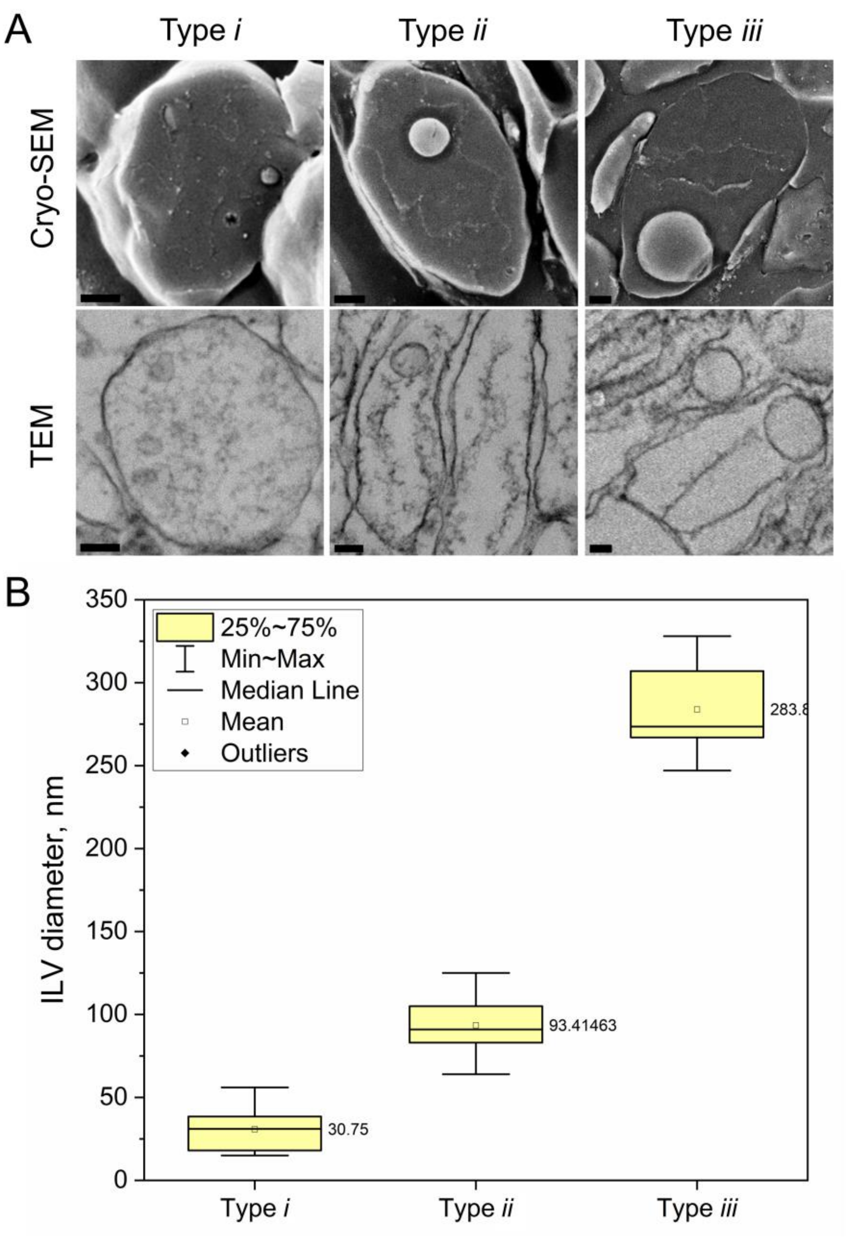

Supplementary Fig. 5. Classification of intraluminal vesicles (ILVs) types observed in forming iridosomes. (A) Cryo-SEM and TEM representative images, and (B) size distribution of each ILV type, n = 60 ILVs from independent iridosomes. Type *i* ILVs are between 25-50 nm in diameter and are associated with early spherical iridosomes and most probably have a role in fibril formation. Type *ii* ILVs are between 80-100 nm in diameter and are usually seen after fibril organization closely associating with one of the fibrils. Type *iii* ILVs are 250-325 nm in diameter and are seen after fibril formation near the iridosome membrane. Scale bars: 100 nm.

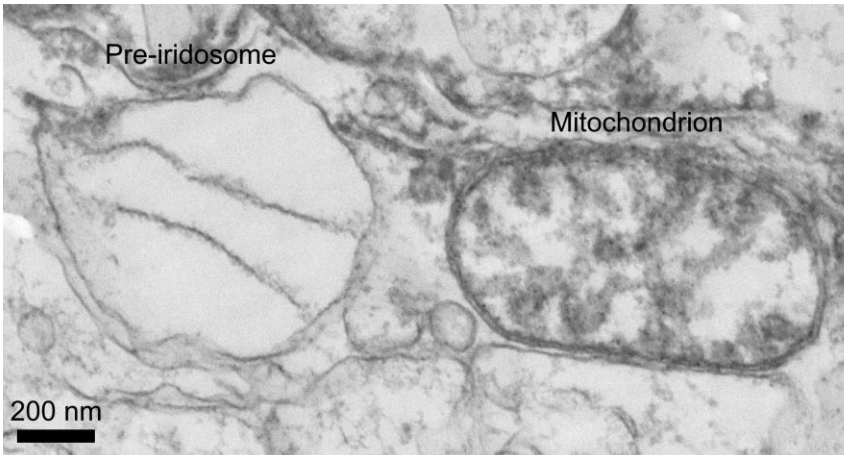

Supplementary Fig. 6. SEM image taken with a STEM detector of a pre-iridosome alongside a mitochondrion showing the origin of “double-membrane” iridosomes. Our observations not only describe the organellogenesis of iridosomes, but also clarify the origin of the “double-membrane” documented previously in these organelles[2-8]. The double-membrane was thought to derive either directly from the ER[4,7] or alternatively from fusion or incorporation of Golgi-derived vesicles with ER-derived vesicles[3,8]. Here we show the observed “inner membrane” is actually formed by the intraluminal sheets that template the crystal growth.

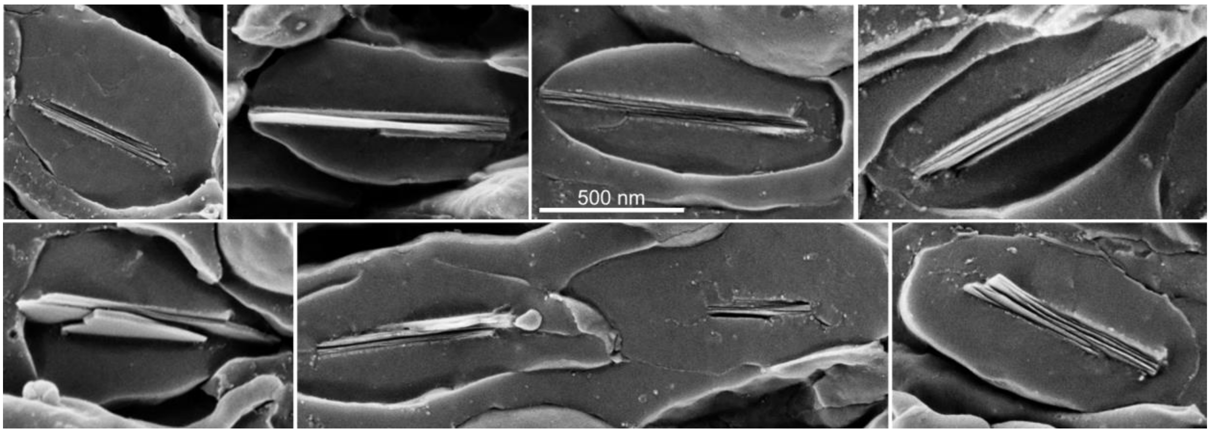

Supplementary Fig. 7. Cryo-SEM images of immature crystals with layered texture. The H-bonded guanine layers are oriented parallel to the (100) face of the crystal (parallel to the H-bonded plane). Initially each layer is 10-13 nm thick. As the crystal matures the layers coalesce to form thicker (25 nm) layers. Scale bar applies to all panels.

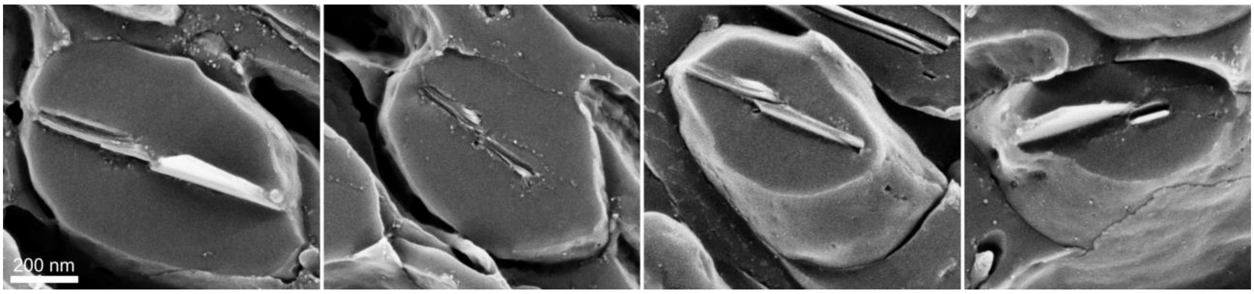

Supplementary Fig. 8. Cryo-SEM images of crystals with multiple nucleation points. The cryo-SEM images indicate that some guanine crystals have multiple nucleation points along the intraluminal sheets. Eventually the separately-nucleated crystals merge to form a single crystal. Scale bar applies to all panels**.**

Supplementary References

1. A. Hirsch et al., “Guanigma”: The revised structure of biogenic anhydrous guanine. *Chem. Mater.* **27**, 8289–8297 (2015). doi:10.1021/acs.chemmater.5b03549.

2. D. Gur, B. A. Palmer, S. Weiner, L. Addadi, Light manipulation by guanine crystals in organisms: Biogenic scatterers, mirrors, multilayer reflectors and photonic crystals. *Adv. Funct. Mater.* **27**, 1603514 (2017). doi: 10.1002/adfm.201603514.

3. R. L. Morrison, S. K. Frost-Mason, Ultrastructural analysis of iridophore organellogenesis in a lizard, Sceloporus graciosus (Reptilia: Phrynosomatidae). *J. Morphol.* **209**, 229–239 (1991). doi:10.1002/jmor.1052090209.

4. J. T. Bagnara et al., Common origin of pigment cells. *Science* **203**, 410–415 (1979). doi:10.1126/science.760198.

5. D. Taylor, The Effects of lntermedin of Amphibian on the Ultrastructure lridophores. *Gen. Comp. Endocrinol.* **12**, 405–416 (1969). doi:10.1016/0016-6480(69)90157-9.

6. K. A. Seitz, Elektronenmikroskopische untersuchungen an den Guanin-Speicherzellen von Araneus diadematus clerck (Araneae, Araneidae). *Zeitschrift für Morphol. der Tiere*. **72**, 245–262 (1972). doi: 10.1007/BF00391554.

7. Y. Kamishima, Electron microscopic study on reflecting platelets in the dorsal iridophores of the sand eel , ammodytes personatus girard. *Proc. Japan Acad*. **54**, 634–639 (1978). doi: 10.2183/pjab.54.634.

8. R. E. Gundersen, R. Rivera, An Ultrastructural Study of the Development of the Dermal lridophores and Structural Pigmentation in Poecilia reticulata. *J. Morphol.* **172**, 349–359 (1982). doi:10.1364/AO.30.001369.
