# Supplementary material for: Macromolecular sheets direct the morphology and orientation of plate-like biogenic guanine crystals
Source: Nat Commun. 2023 Feb 3;14:589. doi: 10.1038/s41467-023-35894-6 (PMC9898273; doi:10.1038/s41467-023-35894-6)
Supplement: Supplementary file 3 — Description of Additional Supplementary Files [file 41467_2023_35894_MOESM3_ESM.pdf]

Description of Additional Supplementary Files

File Name: Supplementary Movie 1

Description: TEM tomography movie of the iridosome shown in Fig. 4b. It is evident that the two intraluminal ‘fibrils’ are in fact 2D sheets which ‘cap’ the (100) faces of the growing guanine crystal.

File Name: Supplementary Movie 2

Description: TEM tomography movie showing several iridosomes at different stages of development in a juvenile scallop eye (~200 µm in diameter).
