# Supplementary material for: Macromolecular sheets direct the morphology and orientation of plate-like biogenic guanine crystals
Source: Nat Commun. 2023 Feb 3;14:589. doi: 10.1038/s41467-023-35894-6 (PMC9898273; doi:10.1038/s41467-023-35894-6)
Supplement: Supplementary file 6 — Reporting Summary [file 41467_2023_35894_MOESM6_ESM.pdf]

nature portfolio

Corresponding author(s): Benjamin A. Palmer

Last updated by author(s): Dec 28, 2022

Reporting Summary

Nature Portfolio wishes to improve the reproducibility of the work that we publish. This form provides structure for consistency and transparency in reporting. For further information on Nature Portfolio policies, see our Editorial Policies and the Editorial Policy Checklist.

Statistics

For all statistical analyses, confirm that the following items are present in the figure legend, table legend, main text, or Methods section.

n/a	Confirmed	
☐	☒	The exact sample size (*n*) for each experimental group/condition, given as a discrete number and unit of measurement
☐	☒	A statement on whether measurements were taken from distinct samples or whether the same sample was measured repeatedly
☒	☐	The statistical test(s) used AND whether they are one- or two-sided<br>*Only common tests should be described solely by name; describe more complex techniques in the Methods section.*
☒	☐	A description of all covariates tested
☒	☐	A description of any assumptions or corrections, such as tests of normality and adjustment for multiple comparisons
☐	☒	A full description of the statistical parameters including central tendency (e.g. means) or other basic estimates (e.g. regression coefficient) AND variation (e.g. standard deviation) or associated estimates of uncertainty (e.g. confidence intervals)
☒	☐	For null hypothesis testing, the test statistic (e.g. *F*, *t*, *r*) with confidence intervals, effect sizes, degrees of freedom and *P* value noted<br>*Give P values as exact values whenever suitable.*
☒	☐	For Bayesian analysis, information on the choice of priors and Markov chain Monte Carlo settings
☒	☐	For hierarchical and complex designs, identification of the appropriate level for tests and full reporting of outcomes
☒	☐	Estimates of effect sizes (e.g. Cohen's *d*, Pearson's *r*), indicating how they were calculated

Our web collection on statistics for biologists contains articles on many of the points above.

Software and code

Policy information about availability of computer code

Data collection	no software was used
Data analysis	FIJI ImageJ 1.52, IMOD 4.11

For manuscripts utilizing custom algorithms or software that are central to the research but not yet described in published literature, software must be made available to editors and reviewers. We strongly encourage code deposition in a community repository (e.g. GitHub). See the Nature Portfolio guidelines for submitting code & software for further information.

Data

Policy information about availability of data

All manuscripts must include a data availability statement. This statement should provide the following information, where applicable:

- Accession codes, unique identifiers, or web links for publicly available datasets
- A description of any restrictions on data availability
- For clinical datasets or third party data, please ensure that the statement adheres to our policy

All the data that support the findings of this study are available from the corresponding author upon request. The data reported in this paper are based on SEM and TEM images which are all shown in an unmodified state in the paper and SI. We would be happy to share uncropped and unannotated raw image files upon request.

Human research participants

Policy information about studies involving human research participants and Sex and Gender in Research.

Reporting on sex and gender	n/a
Population characteristics	n/a
Recruitment	n/a
Ethics oversight	n/a

Note that full information on the approval of the study protocol must also be provided in the manuscript.

Field-specific reporting

Please select the one below that is the best fit for your research. If you are not sure, read the appropriate sections before making your selection.

☒ Life sciences ☐ Behavioural & social sciences ☐ Ecological, evolutionary & environmental sciences

For a reference copy of the document with all sections, see nature.com/documents/nr-reporting-summary-flat.pdf

Life sciences study design

All studies must disclose on these points even when the disclosure is negative.

Sample size	We invesigated 28 eyes, each of the eyes is fractured prior to imaging. Depending on the resolution, each fracture will reveal many thousands of individual vesicles for examination. This provides us with dozens to hundreds of vesicles at each formation stage to image, which is sufficient to obtain an objective picture of iridosome morphogenesis. This information is included in the Methods section.
Data exclusions	no data was excluded
Replication	We invesigated 28 eyes which are effectively 28 replicates. All these eyes contain mirrors with developing iridosomes. The diffrence between them is only in the area exposed for imaging by the random fracture plane (inherent to cryo-SEM method, see Methods section).
Randomization	samples were catagorized by size
Blinding	Blinding is not typically used in such structural biology imaging work. Each of the developing scallops studied has iridophores containing a range of iridosome formation states. By following the crystal progression and growth we were able propose a morphogenetic sequence for these states.

Reporting for specific materials, systems and methods

We require information from authors about some types of materials, experimental systems and methods used in many studies. Here, indicate whether each material, system or method listed is relevant to your study. If you are not sure if a list item applies to your research, read the appropriate section before selecting a response.

Materials & experimental systems

n/a	Involved in the study	
☒	☐	Antibodies
☒	☐	Eukaryotic cell lines
☒	☐	Palaeontology and archaeology
☐	☒	Animals and other organisms
☒	☐	Clinical data
☒	☐	Dual use research of concern

Methods

n/a	Involved in the study	
☒	☐	ChIP-seq
☒	☐	Flow cytometry
☒	☐	MRI-based neuroimaging

Animals and other research organisms

Policy information about studies involving animals; ARRIVE guidelines recommended for reporting animal research, and Sex and Gender in Research

Laboratory animals	No cell lines or laboratory animals were used or involved in this study
Wild animals	did not involve wild animals, the study used commercially available scallops purchased from Scalpro AS as explained in the Materials and Methods section
Reporting on sex	n/a
Field-collected samples	study did not involve samples collected from the field
Ethics oversight	no ethics approval was required as the work only involves molluscs

Note that full information on the approval of the study protocol must also be provided in the manuscript.
